# Mobile phone addiction and depression among Chinese medical students: the mediating role of sleep quality and the moderating role of peer relationships

**DOI:** 10.1186/s12888-022-04183-9

**Published:** 2022-08-23

**Authors:** Ziyi Feng, Yucong Diao, Hongfei Ma, Minghui Liu, Meijun Long, Shuang Zhao, Hui Wu, Yang Wang

**Affiliations:** Department of Social Medicine, College of Health Management, China Medical University, No. 77 Puhe Road, Shenyang North New District, Shenyang, Liaoning 110122 PR China

**Keywords:** Mobile phone addiction, Depression, Peer relationships, Sleep quality, Medical students

## Abstract

The literature has shown that mobile phone addiction is an important risk factor for depression. However, the internal mechanisms of mobile phone addiction leading to depression are still not clear. This study examined the mediating role of sleep quality and moderating role of peer relationships in the association between mobile phone addiction and depression. A sample of 450 Chinese medical students were recruited to complete measures of mobile phone addiction, depression, sleep quality and peer relationships. In this study, SPSS 25.0 and macro PROCESS were used to conduct statistical analysis on the collected data. The results showed that sleep quality partially mediated the association between mobile phone addiction and depression. Moreover, the effect of sleep quality on depression was moderated by peer relationships. The present study can advance our understanding of how and when mobile phone addiction leads to depression. Limitations and implications of this study are discussed.

## Introduction

In June 2020, China’s internet users reached 940 million, including 932 million mobile internet users [1]. As a representative of scientific and technological progress, mobile phones enhance convenience. In terms of information exchange, convenient payment and knowledge acquisition, smartphones improve efficiency. Therefore, smartphones are gradually replacing personal computers and becoming the optimal way for people to access networks [2]. Although smartphones have brought many benefits, the attendant problem of overuse cannot be ignored. “Mobile phone addiction” refers to the excessive dependence on mobile phones in daily life while engaged in other activities, such as studying, partying, and even driving [3]. Researchers using various terms to describe the uncontrolled use of phones, including “excessive use of mobile phones” and “mobile phone dependence” [4]. In this article, the term “mobile phone addiction” is used. This concept is derived from Internet addiction and is mainly classified as compulsive behavior and addiction. More than any other term, mobile phone addiction is associated with physical and psychological dependence, leading to withdrawal symptoms [5]. Mobile phone addiction adversely affects one’s physical and mental health, as well as one’s social functions [6]. The global prevalence rate of mobile phone addiction is 28.3% [7]. Compared with other adults, mobile phone addiction has a very high incidence among young college students [8]. The prevalence of mobile phone addiction among Asian medical students was as high as 41.93% [9]. Studies have shown that medical students mainly suffer from academic pressure because of too many courses [10]. Meanwhile, high academic pressure is also associated with mobile phone addiction [2]. A lot of research has been conducted on the problem of mobile phone addiction among medical students. As medical students become medical professionals after graduation, they face great career and academic pressure [11]. When future medical staff have mental health problems, the quality of medical work may decline, and the doctor–patient relationship can be adversely affected [12]. Therefore, it is necessary to pay more attention to the mental health of medical students. Studies have clarified that mobile phone addiction is correlated with various issues, such as headache [13], impaired vision, and sleep deprivation [14]. In addition, studies have shown an association between mobile phone addiction and depression [15]. However, the internal mechanisms of mobile phone addiction leading to depression in medical students are still not clear, so it proves necessary to analyze these internal mechanisms and provide referable intervention measures to relieve depression in medical students.

### The association between mobile phone addiction and depression

With the continuous development of mobile phone technology, the phenomenon of mobile phone addiction has become problematic [16]. Like internet addiction, mobile phone addiction changes our lives in various ways. Mobile phone addiction entails serious negative effects, including lowering the quality of college students’ interpersonal relationships, leading to loneliness [17]. These negative factors can cause depression among college students [18]. A previous study showed that as a part of internet addiction, mobile phone addiction presented a significant positive correlation with depression [19]. According to a literature review, mobile phone addiction can lead to a series of negative effects in medical students, such as distraction in class, depression or anxiety [20]. In addition, from the perspective of time displacement theory, mobile phone addiction may lead to a decline in social skills and the development of depression [21]. Time displacement [22], in sociology, refers to the idea that new forms of activities may lead to the decline of previously more common activities, such as socializing, work, and even personal care. However, little research has been done on understanding the internal mechanisms between phone addiction and depression. Therefore, this study explores the correlation between medical school students’ mobile phone addiction and depression through analyzing these internal mechanisms.

### The mediating effect of sleep quality

Mobile phone addiction may interfere with students sleep quality. Sleep quality is an important indicator to judge quality of sleep, which is closely related to health. Good sleep quality promotes cognitive function and central nervous system development [23]. In contrast, once sleep disorders occur, they likely affect living conditions, resulting in depression, anxiety and other problems [24]. A study conducted by Yoon and colleagues (2021) showed that smartphone addiction can seriously affect sleep duration [25]. Quite a few students use social media frequently at night, even after midnight, instead of sleeping [26]. Exposure to light from a phone screen suppresses the production of melatonin [27]. The pineal melatonin secretion rhythm affects the mechanism of the sleep/wake cycle [28]. A study of Inkelis and colleagues (2021) showed there was a significant correlation between the sleep quality of students and depression [29]. Insomnia aggravates individual depression and influences medical students’ normal life, study and interpersonal communications. According to Maslow’s hierarchical theory of needs, when sleep needs are difficult to meet, mental and psychological states are affected [30]. Therefore, it is assumed that sleep quality plays a mediating role in the association between mobile phone addiction and depression.

### The moderating effect of peer relationships

In addition, peer relationships can also affect one’s psychological state, to a certain extent. Peer relationships are an important component of interpersonal relationships. It refers to the interpersonal relationship formed and developed in the process of communication between people of similar age or at a similar level of psychological development. Informal social control theory [31] indicates that peer relationships are an informal control source and may have an effect on an individual’s mood and behavior. As children and teenagers grow up, peer relationships become increasingly influential [32]. In this study, peer relationships moderated the association between smartphone addiction and self-evaluation. More specifically, the effect of smartphone addiction on self-evaluation was only significant among students with poor peer relationships [33]. Furthermore, a good peer relationship can enable youth to gain a sense of intimacy and can also relieve the negative influences sometimes caused by family. A previous study supported that teens with good peer relationships had significant differences in terms of depression evaluation compared with teens with poor peer relationships [34]. However, to date, most studies have not considered the effect of peer relationships on mobile phone addiction, sleep quality and depression. Therefore, it is speculated that peer relationships can moderate the impact of mobile phone addiction on mental health. The following hypotheses are proposed:

- **Hypothesis 1.** Sleep quality mediates the association between mobile phone addiction and depression.
- **Hypothesis 2.** The mediating effect of sleep quality on the association between mobile phone addiction and depression is moderated by peer relationships.

**Fig. 1:**
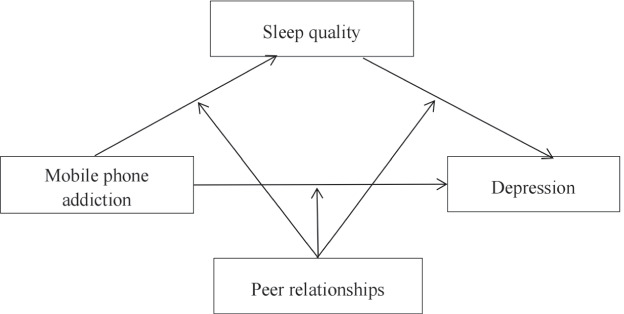
Hypothesis model Annotation: This hypothetical model consists of two parts: Hypothesis 1. Sleep quality mediates the association between mobile phone addiction and depression. Hypothesis 2. The mediating effect of sleep quality on the association between mobile phone addiction and depression is moderated by peer relationships. Hypothesis 2 includes: 1. Peer relationships moderate the effect of mobile phone addiction on depression. 2. Peer relationships moderate the effect of mobile phone addiction on sleep quality. 3. Peer relationships moderate the effect of sleep quality on depression

In conclusion, this study aims to explore the association between medical students’ mobile phone addiction and depression. To reduce the negative effects of medical students’ mobile phone addiction and depression level, this study analyzed the possible mediating role of sleep quality and the moderating role of peer relationships. The hypothetical model is shown in Fig. 1.

## Method

### Participants

This study was ethically approved by the Human Experiment Committee of China Medical University, and all research processes were in line with ethical standards. This study investigated 593 sophomore students from one medical university and collected their mobile phone addiction, depression, peer relationships and demographic variables. The study conducted online surveys during class breaks through the Questionnaire App Wenjuanxing. All questionnaire items were completed in a comprehensible Chinese version, which took approximately 10 minutes. Participants in this study met the following criteria for inclusion: able to read and write Chinese; able to use WeChat to complete the questionnaire independently; and willingness to participate. All the participants provided signed, web-based informed consent before the survey. Participants who took less than 5 min were excluded from the study. After obtaining the data, we screened out 450 valid questionnaires that took more than 5 min to answer, with an effective response rate of 75%.

### Measures

#### Demographic variables and mobile phone usage

Previous studies found that gender [35], major [36], whether the subject was an only child [37], family income per month, cost of living per month [38], residential area of the family [39], types of family [40], and whether the class cadre [41] were all closely related to the main variables in this study. Therefore, they were included as demographic variables in the present study. The number of years of mobile internet access, time spent on the phone every day, and monthly mobile phone consumption were included as mobile phone usage factors in the study.

#### Mobile phone addiction

The Chinese version of the mobile phone addiction index (MPAI) [42] was used to assess the status of mobile phone addiction in medical students. The MPAI, which has cross-gender equivalence among college students [43], has been widely used in the measurement of mobile phone addiction [44]. Participants answered 17 items on a 5-point rating scale that ranges from “1 = not at all” to “5 = always”. The scores were averaged for each question. A higher average score indicated more serious mobile phone addiction. The Cronbach’s alpha of this scale in this study was 0. 90.

#### Depression

The Patient Health Questionnaire 9 (PHQ-9) was developed by Kroenke and colleagues (2001), and is a screening scale commonly used in clinical depression diagnosis and general population research [45]. The scale contains a total of 9 items and uses a 4-point Likert scale. The scores for each question are added up to obtain a total score. The higher the score, the more severe the depression. The Chinese version of the PHQ-9 scale is widely used in China and has good reliability and validity [46]. The Cronbach’s alpha of this scale in this study was 0. 90.

#### Sleep quality

The Pittsburgh Sleep Quality Index (PSQI) compiled by Buysse and colleagues (1989) was used for the evaluation of sleep quality in patients with sleep disorders and mental disorders, as well as a self-rating scale for the evaluation of sleep quality in the general human population [47]. The Chinese version of the scale also has good reliability and validity among medical students [48]. Participants responded to 18 items by which seven aspects of sleep quality were assessed (e.g., sleep latency, sleep persistence, and sleep disorders). Each component was scored according to the 0–3 grade, and the cumulative score of each component was the total score of the PSQI, which ranged from 0 to 21. The higher the score, the worse the sleep quality. The Cronbach’s alpha of this scale in this study was 0. 84.

#### Peer relationships

The questionnaire used to measure peer relationships was the Inventory of Parent and Peer Attachment (IPPA) compiled by Armsden and Greenberg [49]. The scale is divided into three parts: father attachment, mother attachment and peer attachment. The peer attachment scale was used in this study. Participants needed to answer 25 items on a 5-point rating scale that ranged from “1 = not at all” to “5 = always”. The scores were added up for each question to obtain a total score. Furthermore, the Chinese version of the scale has good reliability and validity [50]. Higher scores indicate higher levels of peer relationships. The Cronbach’s alpha of this scale in this study was 0.85.

### Statistical analysis

In this study, SPSS 25.0 and GPower was used to conduct statistical analysis on the collected data. First, Gpower was used to conduct power analyses. Next, a t-test and ANOVA were used to test differences among the group in demographic variables and mobile phone usage. Pearson correlation was used to test the bivariate correlation of all the study variables. Finally, the SPSS25 macro PROCESS (Models 4 and 59) proposed by Hayes was used to test the proposed model [51].

## Results

### Preliminary analyses

The sample size calculation equation is as follows: $n=\left(\frac{u_a^2\pi \left(1-\pi \right)}{\delta^2}\right)$.

The detection rate of depression among college students in China was π = 37% [52]. A relative error of 15% was allowed in the present study. The absolute error can be calculated by δ = 0.15π = 0.15 × 37%. We adopt 95% confidence intervals; thus, μ_a_ = 1.96. According to the following equation for the sample size, we calculated the minimum sample size: n = [1.962 × 37% × (1–37%)]/(0.15 × 37%)^2^ ≈ 296. Considering the pass rate of questionnaire, the desired sample size should increase by 10%: 296 × (1 + 10%) ≈ 325. The final sample size of this study is 450. We conducted power analyses of the sample size through GPower. When α = 0. 05, effect size = 0.3, the power of this sample size was 0.99. Among the 450 students who participated in the study, 174 were male, accounting for 38.7% of the total participants, and 276 were female, accounting for 61.3% of the total participants. Clinical majors accounted for 84.9% of total participants, and the only child accounted for 56.2% of total participants. Other demographic variables and mobile phone usage of participants are shown in Table 1. Being an only child, monthly family income, the area where the family lives, and time spent on the phone every day were significantly associated with depression scores.

**Table 1:** Demographic variables and mobile phone usage

Variables	n	%	*P*
**Gender**			0.754
Male	174	38.7	
Female	276	61.3	
**Major**			0.278
Clinical medicine	382	84.9	
No clinical medicine	68	15.1	
**Only child**			0.000***
Yes	253	56.2	
No	197	43.8	
**Family income per month**			0.050*
<3000 RMB	32	7	
3000–6000 RMB	120	26.7	
6000–9000 RMB	114	25.3	
>9000 RMB	184	40.9	
**Cost of living per month**			0.244
<1000 RMB	37	8.2	
1000–1500 RMB	176	39.1	
1500–2000 RMB	170	37.8	
>2000 RMB	67	14.9	
**Family area**			0.001**
Cities and towns	341	75.8	
Countryside	109	24.2	
**Types of family**			0.926
Complete family	395	87.8	
Incomplete family	55	12.2	
**Class cadre**			0.982
Yes	169	37.6	
No	281	62.4	
**The number of years of mobile internet access**			0.13
< 2 years	47	10.4	
2–4 years	129	28.7	
> 4 years	274	60.9	
**Time spent on the phone every day**			0.000***
< 4 hours	82	18.2	
4–6 hours	191	42.4	
> 6 hours	177	39.3	
**Monthly mobile phone consumption**			0.561
< 30 RMB	49	10.9	
30–50 RMB	179	39.8	
50–100 RMB	123	27.3	
100–150 RMB	38	8.4	
> 150 RMB	61	13.6	

^*^p < 0.05

^**^p < 0.01

^***^p < 0.001

Table 2 shows the correlations of all observed variables, as well as their mean and standard deviation. The mean score of the PHQ-9 was 5.09 (SD = 4.48). The mean MPAI score was 2.57 (SD = 0.72). The mean PSQI and IPPA scores were 4.18 (SD = 2.86) and 47.02 (SD = 11.98), respectively. As hypothesized, depression was positively correlated with mobile phone addiction (*r* = 0.37, *p* < 0.01) and poor sleep quality (*r* = 0.62, *p* < 0.01) and negatively correlated with peer relationships (*r* = − 0.37, *p* < 0.01). Mobile phone addiction was positively correlated with poor sleep quality (*r* = 0.27, *p* = 0.01) and negatively associated with peer relationships (*r* = − 0.14, *p* < 0.01). Poor sleep quality was negatively correlated with peer relationships (*r* = − 0.24, *p* < 0.01).

**Table 2:** Descriptive statistics and intercorrelations between variables

Variable	M	SD	Depression	Mobile phone addiction	Poor sleep quality	Peer relationships
Depression	5.09	4.48	1.00			
Mobile phone addiction	2.57	0.72	0.37^a^	1.00		
Poor sleep quality	4.18	2.86	0.62^a^	0.27^a^	1.00	
Peer relationships	47.02	11.98	−0.37^a^	−0.14^a^	-0.24^a^	1.00

^a^Correlation is significant at the 0.01 level (2-tailed)

### Mediation model testing

Model 4 of PROCESS macro was adapted to test if sleep quality mediated the association between mobile phone addiction and depression under the conditions of controlling “only child, family income per area, family area and time spend on the phone every day”. As shown in Table 3 Model 1, the predictive effect of mobile phone addiction on depression was significant (β = 0.34, *p* < 0.001). As shown in Table 3 Model 2, mobile phone addiction had a significant positive predictive effect on poor sleep quality (β = 0.22, *p* < 0.001). As shown in Table 3 Model 3，poor sleep quality also had a significant positive predictive effect on depression (β = 0.49, *p* < 0.001). By bootstrapping 5000 samples, the 95% confidence intervals of the direct effect of mobile phone addiction on depression and the mediating effect of poor sleep quality did not contain 0, which indicated that mobile phone addiction not only directly predicted depression, but also predicted depression through the mediating effect of poor sleep quality. Therefore, H1 was supported.

**Table 3:** Conditional process analysis

Model						
Model 1: The total effect model (criterion: depression)						
R	R2	F	P	β	t	P
0.41	0.17	18.55	< 0.001			
Constant				−0.49	−1.80	0.07
Only child				0.25^**^	2.69	< 0.01
Family income per month				−0.04	− 0.99	0.32
Family area				0.08	0.70	0.48
Time spent on the phone every day				0.07	1.24	0.21
Mobile phone addiction				0.34^***^	7.42	< 0.001
Model 2: Mediator variable model (criterion: poor sleep quality)						
R	R2	F	P	β	t	P
0.36	0.13	9.51	< 0.001			
Constant				−0.41	−1.46	0.14
Only child				0.14	1.52	0.12
Family income per month				0.08	1.72	0.08
Family area				−0.11	−0.94	0.34
Time spent on the phone every day				0.04	0.62	0.53
Mobile phone addiction				0.22^***^	4.81	< 0.001
Peer relationships				−0.21^***^	−4.75	< 0.001
Mobile phone addiction x Peer relationships				0.04	1.08	0.27
Model 3: Dependent variable model (criterion: depression)						
R	R2	F	P	β	t	P
0.71	0.50	50.81	< 0.001			
Constant				−0.49^*^	−2.31	0.02
Only child				0.17^*^	2.32	0.02
Family income per month				−0.02	−0.65	0.51
Family area				0.14^*^	1.58	0.11
Time spent on the phone every day				0.05	1.03	0.30
Mobile phone addiction				0.20^***^	5.44	< 0.001
Poor sleep quality				0.49^***^	13.74	< 0.001
Peer relationships				−0.21^***^	−5.96	< 0.001
Poor sleep quality x Peer relationships				−0.11^**^	−3.11	0.002
Mobile phone addiction x Peer relationships				−0.03	−1.15	0.24

*N* = 450

^*^p < 0.05

^**^p < 0.01

^***^p < 0.001

### Moderated mediation model testing

**Fig. 2:**
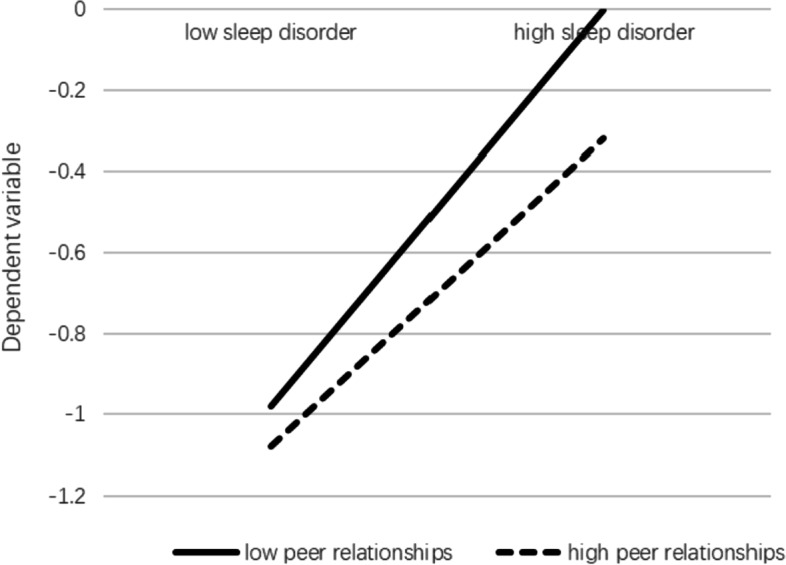
Peer relationships moderate the relation between sleep quality and depression

The moderating effect of peer relationships in this mediating role can be seen in Table 3. When peer relationships were included in the model, the interaction of poor sleep quality and peer relationships was significant predictors of depression (β = − 0.11, *p* < 0.01), which indicated that peer relationships can regulate the predictive effect of poor sleep quality on depression. In addition, the interaction of mobile phone addiction and peer relationships showed no significant effects on poor sleep quality (β = 0.04, *p* = 0.27) or depression (β = -0.03, *p* = 0.24). This suggested that peer relationships did not moderate the pathway of “mobile phone addiction-sleep quality” or the direct pathway of “mobile phone addiction-depression” to anxiety. Therefore, H2 was partly supported. Further, simple slope analysis (Fig. 2) showed that for subjects with a low peer relationship level (M-1SD), poor sleep quality had a significant positive predictive effect on depression. For subjects with higher peer relationship levels (M + 1SD), poor sleep quality also had a positive predictive effect on depression, but its predictive effect was small, indicating that the predictive effect of poor sleep quality on depression gradually decreased with the improvement of individual peer relationships.

## Discussion

In this study, a moderated mediation model was established to explore the association between mobile phone addiction and depression in Chinese medical students. The mean depression score was 5.09. According to the PHQ-9 scale, a score between 5 ~ 9 is classified as mild depression [45]. The mean score of mobile phone addiction of medical students was 2.57, higher than the median score of 2.37 of college students [53]. Therefore, depression and mobile phone addiction of medical students needs more attention. The results of the moderated mediation model showed that sleep quality played a mediating role in the association between mobile phone addiction and depression. At the same time, peer relationships moderated the association between sleep quality and depression.

### Association between mobile phone addiction and depression

First, it was found that mobile phone addiction was a significant predictor of depression in medical students, which was consistent with many existing studies [54]. Students who are dependent on mobile phones tend to spend more time on them, lack sufficient and effective communication with classmates and teachers around them, and may even have family conflicts, all of which make it more likely for them to suffer from depression and other psychological problems [55]. Medical students who are recently living independently from their parents on campus, may lack the self-discipline needed to moderate mobile phone usage. They are more likely to obtain pleasure and satisfaction from their mobile phones than to actively participate in various sports and sports activities. Long-term addiction to mobile phones can cause medical students to become disassociated from real life, have negative thoughts, and even lead to depression and sleep disorders [15]. The results of this study support the perspective of time displacement theory. The great contrast between the virtual world of the internet and the real world makes it difficult for students who are addicted to mobile phones to strike a balance.

### Mediating effect of sleep quality

The internal mechanisms of depression caused by mobile phone addiction cannot be ignored. This study showed that poor sleep quality has a partial mediating effect between mobile phone addiction and depression, which is consistent with the results of the literature [56]. Numerous studies have also shown that mobile phone addiction significantly affects sleep quality [57]. As medical students have a full course schedule in the daytime, they do not have much time to use mobile phones [58]. As a result, more time before bed at night is spent on the phone. The intense stimulation brought by the mobile phone network makes it difficult for students to fall asleep immediately after putting down the mobile phone [59]. The emission of blue light from the mobile phone screen also interferes with the circadian rhythm and affects sleep hygiene. For healthy humans, the decrease in sleep quality makes it difficult to have enough energy for the following day’s work and study requirements, which leads to a significant decrease in the efficiency of work. At the same time, due to poor sleep quality and the physical and psychological effects of this, some students are even more prone to depression, anxiety and other negative emotions [60].

### Moderating effect of peer relationships

It was found that peer relationships moderated the relationship between sleep quality and depression. Previous literature has shown that peer relationships can influence the mental health of students to some extent [61], but no relevant literature has studied the moderating effect of peer relationships on the association between sleep quality and depression. This regulating effect can be explained by the following reasons. First, students who live far away from their parents in college accommodation spend most of their time with peers, resulting in peers being more in tune the real life of medical students than parents and teachers [62]. Good interpersonal relationships can bring a sense of security, and students can obtain a sense of happiness from the company of others [63]. Therefore, students with better peer relationships are more likely to have better sleep quality [62], and thus, a lower risk of depression. Second, medical students are more inclined to share various things happening in their lives with their peers, including when they feel pressure or are confused [64]. It can be inferred that students with good peer relationships release stress effectively through talking, thus reducing the probability of depression [65]. Moreover, students with good peer relationships have higher psychological resiliency [66] in the face of sleep disorders. They have a greater ability to self-regulate and minimize the negative effects of sleep disturbances. Thus, the quality of peer relationships can effectively regulate the association between sleep quality and depression.

## Limitations and implications

Some limitations must be considered when interpreting the findings of this study. First, this study only collected questionnaires from sophomores of a particular medical school, thus the results of this study are not generalized. Second, all the questionnaires in this study are from individuals’ self-reports. In the future, data from multiple perspectives, such as teachers, parents and peers, should be collected. Third, other interpersonal relationships, such as teacher-student relationships and romantic relationships, may also influence medical students’ depression. Fourth, this study is a cross-sectional study and cannot be used to infer causality.

Although this study has some limitations, the findings of this study are still of great significance. First, the findings underscore the importance of mobile phone addiction in predicting depression in Chinese medical students. Considering a series of negative effects of depression on medical students, such as decreased academic performance and a sense of self-efficacy, paying attention to the degree of students’ mobile phone addiction is beneficial to reduce the probability of depression [67]. Second, by establishing a mediation model, the research results can help explain the association between mobile phone addiction and medical students’ depression, as well as explain the effectiveness of intervention measures. For example, improving sleep quality in students can reduce the risk of depression. Third, interpersonal relationships are the basis of human survival. As an important part of interpersonal relationships, peer relationships have been proven to play a very important role in many studies [68]. It is necessary to recognize the moderating role of peer relationships in the association between sleep quality and students’ depression. Good peer relationships can reduce depression and its negative effects.

According to the results of this paper, we suggest that both universities and society should pay attention to the mental health of medical students, such as setting up enough psychological help institutions in and out of school, so that students can seek help when needed [69]. We also need to focus on the phenomenon of mobile phone addiction and guide school students to form the habit of going to bed early and getting up early to improve the quality of sleep. At the same time, students should be encouraged to devote more time to social interaction and develop better peer relationships. Schools should also actively hold various social activities to promote emotional communication among students. Subsequent studies can expand the sample size to cover a wider population. It can also be expanded in the study of related variables, and other variables such as anxiety and parent-child relationships can also be included in the research scope.

## Data Availability

The datasets generated and/or analysed during the current study are not publicly available due the data also forms part of an ongoing study but are available from the corresponding author on reasonable request.
